# Ethyl *N*-(2-acetyl-3-oxo-1-phenyl­but­yl)carbamate

**DOI:** 10.1107/S1600536813024884

**Published:** 2013-09-12

**Authors:** Alexander N. Volov, Ilia A. Zamilatskov

**Affiliations:** a A. N. Frumkin Institute of Physical Chemistry and Electrochemistry RAS, Leninsky prospect 31, Moscow 119071, Russian Federation

## Abstract

In the title compound, C_15_H_19_NO_4_, all three carbonyl groups are *syn*-oriented with respect to the methine group attached to the phenyl ring. The mean planes of the phenyl ring and ethyl carbamate moiety form a dihedral angle of 65.2 (1)°. In the crystal, mol­ecules related by translation in [100] are linked into chains *via* N—H⋯O hydrogen bonds.

## Related literature

For details of the synthesis, see: Kuzmina *et al.* (2013 ▶). For the crystal structures of related compounds, see: Hatano *et al.* (2008 ▶).
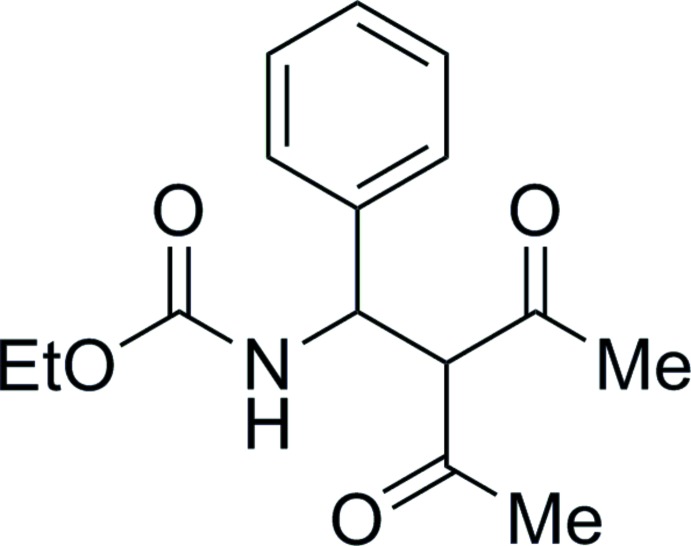


## Experimental

### 

#### Crystal data

C_15_H_19_NO_4_
*M* *_r_* = 277.31
Triclinic, 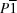

*a* = 5.392 (2) Å
*b* = 9.204 (2) Å
*c* = 15.841 (6) Å
α = 81.58 (2)°
β = 81.98 (2)°
γ = 89.13 (3)°
*V* = 770.1 (4) Å^3^
*Z* = 2
Cu *K*α radiation
μ = 0.71 mm^−1^
*T* = 295 K
0.50 × 0.21 × 0.10 mm

#### Data collection

Enraf–Nonius CAD-4 diffractometer
Absorption correction: ψ scan (North *et al.*, 1968 ▶) *T*_min_ = 0.76, *T*_max_ = 0.92
4976 measured reflections
3176 independent reflections
2057 reflections with *I* > 2σ(*I*)
*R*_int_ = 0.022
2 standard reflections every 150 reflections intensity decay: 3%

#### Refinement

*R*[*F* ^2^ > 2σ(*F* ^2^)] = 0.068
*wR*(*F* ^2^) = 0.231
*S* = 1.04
3176 reflections
189 parameters
15 restraints
H atoms treated by a mixture of independent and constrained refinement
Δρ_max_ = 0.30 e Å^−3^
Δρ_min_ = −0.19 e Å^−3^

### 

Data collection: *CAD-4 EXPRESS* (Enraf–Nonius, 1994 ▶); cell refinement: *CAD-4 EXPRESS*; data reduction: *XCAD4* (Harms & Wocadlo, 1995 ▶); program(s) used to solve structure: *SHELXS97* (Sheldrick, 2008 ▶); program(s) used to refine structure: *SHELXL97* (Sheldrick, 2008 ▶); molecular graphics: *PLATON* (Spek, 2009 ▶) and *Mercury* (Macrae *et al.*, 2006 ▶); software used to prepare material for publication: *SHELXL97*.

## Supplementary Material

Crystal structure: contains datablock(s) I, 1R. DOI: 10.1107/S1600536813024884/rk2414sup1.cif

Structure factors: contains datablock(s) I. DOI: 10.1107/S1600536813024884/rk2414Isup2.hkl

Supplementary material file. DOI: 10.1107/S1600536813024884/rk2414Isup3.cml

Additional supplementary materials:  crystallographic information; 3D view; checkCIF report

## Figures and Tables

**Table 1:** Hydrogen-bond geometry (Å, °)

*D*—H⋯*A*	*D*—H	H⋯*A*	*D*⋯*A*	*D*—H⋯*A*
N1—H1⋯O2^i^	0.97 (3)	2.26 (3)	3.180 (4)	158 (2)

Symmetry code: (i) 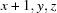.

**Fig. 1.**
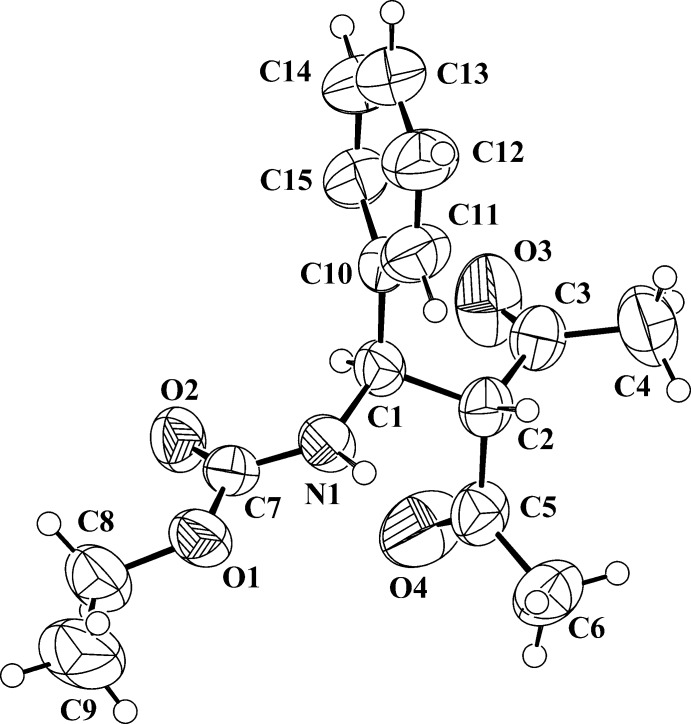
Molecular structure of I whith the atom numbering scheme. Displacement ellipsoids are drawn at 50% probability level. H atoms are presented as a small spheres of arbitrary radius.

**Fig. 2.**
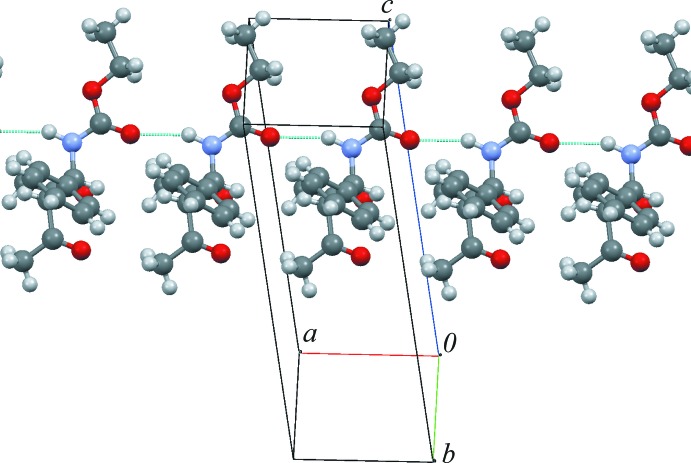
Portion of the crystal packing in I. Dotted blue lines denote hydrogen bonds.

## supplementary crystallographic information

### 1. Comment

Recently, we have developed a simple general five-step approach for the synthesis of macrocycles containing semicarbazide moieties *via* heterocyclization of semicarbazides with oxo group in 3 position (Kuzmina *et al.*, 2013). The title compound, I, has been obtained as an intermediate product. Herewith we present its molecular and crystal structure.

In I (Fig. 1), all bond lengths and angles are normal and correspond well to those observed in the related (1*R*,2*R*)-benzyl (3-oxo-2-((2-oxo-1,3-oxazolidin-3-yl)carbonyl)-1-phenylbutyl)carbamate and (*S*)-benzyl (2-acetyl-1-(4-bromophenyl)-2-hydroxy-3-oxobutyl)carbamate (Hatano *et al.*, 2008). In the molecule, all three carbonyl groups are *syn* oriented with respect to the methine group attached to the phenyl ring.

In the crystal, the molecules related by translation in [1 0 0] are linked into chains *via* intermolecular classical N–H···O hydrogen bonds (Table 1, Fig. 2).

### 2. Experimental

To a solution of KOH (0.277 g, 0.0049 mol) in 30 ml ethanol was added acetylacetone (0.495 g, 0.0049 mol) and stirred vigorously for 10 minutes. Ethyl *N*-[(tosyl)(phenyl)methyl]carbamate (1.5 g, 0.0045 mol) was added and reaction mixture stirred for 6.5 h. After completion of reaction, solution was evaporated to dryness and residue washed with saturated solution of NaHCO_3_, hexane and dried to give 1.1 g (88%) of ethyl *N*-[(2-acetyl-3-oxo-1-phenyl)butyl]carbamate as white powder. An analytically pure sample was obtained by recrystallization from *Me*OH-H_2_O (2:3). M.p. 421-422 K (*Me*OH-H_2_O (2:3)).

^1^H NMR (600 MHz; DMSO-d6): δ_H_, p.p.m. 7.76(1*H*, d, J = 9.16 Hz, NH), 7.21-7.30 (5*H*, m, H in *Ph*), 5.15 (1*H*, t, J = 9.78 Hz, CH), 4.47(1*H*, d, J = 11.12 Hz, CH), 3.87-3.93 (2*H*, m, CH_2_ in COO*Et*), 2.22 (3*H*, s, CH_3_ in acetyl), 1.87 (3*H*, s, CH_3_ in acetyl), 1.08 (3*H*, t, J = 7.04 Hz, CH_3_ in COO*Et*). ^13^C NMR (150 MHz; DMSO-d6): δ_H_, p.p.m. 14.4, 30.1, 30.7, 54.1, 59.9, 71.7, 127.3, 127.5, 128.4, 140.6, 155.3, 201.3, 201.5 Anal. Calcd for C_15_H_19_NO_4_: C, 64.97; H, 6.91; N 5.05. Found: C, 65.09; H, 6.98; N, 5.12.

### 3. Refinement

Atom H1 was located on a difference map and isotropically refined. C-bound H atoms were positioned geometrically (C–H = 0.93Å-0.98Å) and refined as riding, with *U_iso_*(H) = 1.2-1.5*U_eq_*(C).

### Crystal data

**Table:**

C_15_H_19_NO_4_	*F*(000) = 296
*M**_r_* = 277.31	*D*_x_ = 1.196 Mg m^−^^3^
Triclinic, *P*1	Melting point = 421–422 K
*a* = 5.392 (2) Å	Cu *K*α radiation, λ = 1.54184 Å
*b* = 9.204 (2) Å	Cell parameters from 25 reflections
*c* = 15.841 (6) Å	θ = 31.6–34.9°
α = 81.58 (2)°	µ = 0.71 mm^−^^1^
β = 81.98 (2)°	*T* = 295 K
γ = 89.13 (3)°	Prism, colourless
*V* = 770.1 (4) Å^3^	0.50 × 0.21 × 0.10 mm
*Z* = 2	

### Data collection

**Table:**

Enraf–Nonius CAD-4 diffractometer	2057 reflections with *I* > 2σ(*I*)
Radiation source: fine-focus sealed tube	*R*_int_ = 0.022
Graphite monochromator	θ_max_ = 74.9°, θ_min_ = 2.9°
non–profiled ω–scans	*h* = −3→6
Absorption correction: ψ scan (North *et al.*, 1968)	*k* = −11→11
*T*_min_ = 0.76, *T*_max_ = 0.92	*l* = −19→19
4976 measured reflections	2 standard reflections every 150 reflections
3176 independent reflections	intensity decay: 3%

### Refinement

**Table:**

Refinement on *F*^2^	Secondary atom site location: difference Fourier map
Least-squares matrix: full	Hydrogen site location: inferred from neighbouring sites
*R*[*F*^2^ > 2σ(*F*^2^)] = 0.068	H atoms treated by a mixture of independent and constrained refinement
*wR*(*F*^2^) = 0.231	*w* = 1/[σ^2^(*F*_o_^2^) + (0.11*P*)^2^ + 0.24*P*] where *P* = (*F*_o_^2^ + 2*F*_c_^2^)/3
*S* = 1.04	(Δ/σ)_max_ < 0.001
3176 reflections	Δρ_max_ = 0.30 e Å^−^^3^
189 parameters	Δρ_min_ = −0.19 e Å^−^^3^
15 restraints	Extinction correction: *SHELXL97* (Sheldrick, 2008), Fc^*^=kFc[1+0.001xFc^2^λ^3^/sin(2θ)]^-1/4^
Primary atom site location: structure-invariant direct methods	Extinction coefficient: 0.009 (2)

### Special details

**Table:**

Geometry. All s.u.'s (except the s.u. in the dihedral angle between two l.s. planes) are estimated using the full covariance matrix. The cell e.s.d.'s are taken into account individually in the estimation of s.u.'s in distances, angles and torsion angles; correlations between s.u.'s in cell parameters are only used when they are defined by crystal symmetry. An approximate (isotropic) treatment of cell s.u.'s is used for estimating s.u.'s involving l.s. planes.
Refinement. Refinement of *F*^2^ against ALL reflections. The weighted *R*-factor *wR* and goodness of fit *S* are based on *F*^2^, conventional *R*-factors *R* are based on *F*, with *F* set to zero for negative *F*^2^. The threshold expression of *F*^2^ > σ(*F*^2^) is used only for calculating *R*-factors(gt) *etc*. and is not relevant to the choice of reflections for refinement. *R*-factors based on *F*^2^ are statistically about twice as large as those based on *F*, and *R*-factors based on ALL data will be even larger.

### Fractional atomic coordinates and isotropic or equivalent isotropic displacement parameters (Å^2^)

**Table:**

	*x*	*y*	*z*	*U*_iso_*/*U*_eq_	
O1	0.1419 (4)	0.4031 (2)	0.90111 (13)	0.0701 (6)	
O2	−0.1063 (4)	0.5365 (2)	0.81579 (14)	0.0702 (6)	
O3	0.3293 (5)	0.8086 (4)	0.55078 (16)	0.1061 (10)	
O4	0.3031 (5)	0.4665 (3)	0.6140 (3)	0.1242 (12)	
N1	0.3154 (5)	0.5683 (3)	0.79670 (16)	0.0609 (6)	
H1	0.473 (6)	0.541 (3)	0.8167 (19)	0.064 (8)*	
C1	0.3263 (5)	0.6871 (3)	0.72419 (17)	0.0553 (6)	
H1A	0.1667	0.6874	0.7012	0.066*	
C2	0.5335 (5)	0.6570 (3)	0.65270 (18)	0.0581 (7)	
H2	0.6965	0.6633	0.6726	0.070*	
C3	0.5268 (6)	0.7669 (4)	0.57095 (19)	0.0649 (7)	
C4	0.7610 (7)	0.8120 (5)	0.5160 (3)	0.1017 (13)	
H4A	0.7268	0.8494	0.4589	0.153*	
H4B	0.8706	0.7291	0.5137	0.153*	
H4C	0.8396	0.8873	0.5391	0.153*	
C5	0.5017 (6)	0.5036 (4)	0.6283 (2)	0.0691 (8)	
C6	0.7211 (7)	0.4071 (4)	0.6219 (3)	0.0900 (11)	
H6A	0.6725	0.3140	0.6088	0.135*	
H6B	0.7871	0.3930	0.6758	0.135*	
H6C	0.8470	0.4515	0.5770	0.135*	
C7	0.1002 (5)	0.5061 (3)	0.83558 (17)	0.0548 (6)	
C8	−0.0754 (8)	0.3217 (4)	0.9476 (2)	0.0914 (12)	
H8A	−0.1578	0.2732	0.9086	0.110*	
H8B	−0.1940	0.3873	0.9743	0.110*	
C9	0.0159 (12)	0.2109 (5)	1.0147 (3)	0.1292 (19)	
H9A	0.1200	0.1409	0.9873	0.194*	
H9B	−0.1247	0.1611	1.0506	0.194*	
H9C	0.1111	0.2595	1.0493	0.194*	
C10	0.3566 (5)	0.8362 (3)	0.75167 (17)	0.0588 (7)	
C11	0.5396 (6)	0.8614 (3)	0.8002 (2)	0.0796 (9)	
H11	0.6451	0.7851	0.8171	0.096*	
C12	0.5704 (8)	0.9968 (3)	0.8243 (3)	0.0980 (12)	
H12	0.6966	1.0120	0.8566	0.118*	
C13	0.4143 (8)	1.1085 (4)	0.8004 (3)	0.1067 (15)	
H13	0.4308	1.1995	0.8181	0.128*	
C14	0.2344 (9)	1.0878 (4)	0.7509 (3)	0.1055 (14)	
H14	0.1319	1.1652	0.7331	0.127*	
C15	0.2056 (7)	0.9521 (3)	0.7275 (2)	0.0822 (10)	
H15	0.0808	0.9382	0.6944	0.099*	

### Atomic displacement parameters (Å^2^)

**Table:**

	*U*^11^	*U*^22^	*U*^33^	*U*^12^	*U*^13^	*U*^23^
O1	0.0820 (15)	0.0563 (11)	0.0669 (12)	−0.0039 (10)	−0.0069 (10)	0.0051 (9)
O2	0.0492 (11)	0.0760 (13)	0.0806 (13)	−0.0023 (9)	−0.0063 (10)	0.0021 (10)
O3	0.0653 (15)	0.159 (3)	0.0856 (17)	0.0111 (16)	−0.0218 (13)	0.0192 (17)
O4	0.0603 (15)	0.115 (2)	0.220 (4)	−0.0017 (14)	−0.0398 (19)	−0.079 (2)
N1	0.0470 (13)	0.0575 (13)	0.0750 (15)	−0.0004 (10)	−0.0148 (11)	0.0071 (11)
C1	0.0428 (13)	0.0567 (14)	0.0633 (15)	0.0001 (11)	−0.0069 (11)	0.0010 (12)
C2	0.0391 (13)	0.0660 (16)	0.0710 (16)	0.0013 (11)	−0.0118 (12)	−0.0116 (13)
C3	0.0503 (16)	0.0802 (19)	0.0655 (16)	0.0004 (14)	−0.0103 (13)	−0.0128 (14)
C4	0.071 (2)	0.113 (3)	0.108 (3)	−0.014 (2)	0.003 (2)	0.016 (2)
C5	0.0503 (16)	0.0778 (19)	0.083 (2)	−0.0032 (14)	−0.0095 (14)	−0.0224 (16)
C6	0.067 (2)	0.086 (2)	0.125 (3)	0.0126 (18)	−0.016 (2)	−0.039 (2)
C7	0.0602 (17)	0.0465 (13)	0.0571 (14)	−0.0002 (11)	−0.0058 (12)	−0.0076 (11)
C8	0.108 (3)	0.076 (2)	0.080 (2)	−0.017 (2)	0.008 (2)	0.0016 (18)
C9	0.195 (6)	0.083 (3)	0.092 (3)	−0.001 (3)	0.011 (3)	0.017 (2)
C10	0.0480 (14)	0.0566 (15)	0.0660 (16)	0.0041 (11)	0.0049 (12)	−0.0026 (12)
C11	0.070 (2)	0.0651 (18)	0.109 (3)	0.0108 (15)	−0.0193 (18)	−0.0244 (18)
C12	0.091 (3)	0.074 (2)	0.136 (3)	0.002 (2)	−0.016 (2)	−0.039 (2)
C13	0.106 (3)	0.064 (2)	0.142 (4)	0.003 (2)	0.020 (3)	−0.028 (2)
C14	0.107 (3)	0.068 (2)	0.134 (4)	0.028 (2)	−0.002 (3)	−0.006 (2)
C15	0.073 (2)	0.071 (2)	0.097 (2)	0.0179 (17)	−0.0054 (18)	−0.0011 (17)

### Geometric parameters (Å, º)

**Table:**

O1—C7	1.341 (3)	C6—H6B	0.9600
O1—C8	1.450 (4)	C6—H6C	0.9600
O2—C7	1.216 (3)	C8—C9	1.491 (6)
O3—C3	1.196 (4)	C8—H8A	0.9700
O4—C5	1.190 (4)	C8—H8B	0.9700
N1—C7	1.331 (4)	C9—H9A	0.9600
N1—C1	1.461 (3)	C9—H9B	0.9600
N1—H1	0.97 (3)	C9—H9C	0.9600
C1—C10	1.516 (4)	C10—C11	1.374 (3)
C1—C2	1.529 (4)	C10—C15	1.375 (3)
C1—H1A	0.9800	C11—C12	1.375 (3)
C2—C3	1.526 (4)	C11—H11	0.9300
C2—C5	1.536 (4)	C12—C13	1.363 (3)
C2—H2	0.9800	C12—H12	0.9300
C3—C4	1.459 (5)	C13—C14	1.362 (3)
C4—H4A	0.9600	C13—H13	0.9300
C4—H4B	0.9600	C14—C15	1.371 (3)
C4—H4C	0.9600	C14—H14	0.9300
C5—C6	1.469 (5)	C15—H15	0.9300
C6—H6A	0.9600		
			
C7—O1—C8	116.4 (3)	O2—C7—N1	126.0 (3)
C7—N1—C1	122.2 (2)	O2—C7—O1	123.9 (3)
C7—N1—H1	121.8 (18)	N1—C7—O1	110.1 (2)
C1—N1—H1	116.0 (18)	O1—C8—C9	107.0 (4)
N1—C1—C10	112.0 (2)	O1—C8—H8A	110.3
N1—C1—C2	109.9 (2)	C9—C8—H8A	110.3
C10—C1—C2	112.1 (2)	O1—C8—H8B	110.3
N1—C1—H1A	107.6	C9—C8—H8B	110.3
C10—C1—H1A	107.6	H8A—C8—H8B	108.6
C2—C1—H1A	107.6	C8—C9—H9A	109.5
C3—C2—C1	111.4 (2)	C8—C9—H9B	109.5
C3—C2—C5	106.9 (2)	H9A—C9—H9B	109.5
C1—C2—C5	110.7 (2)	C8—C9—H9C	109.5
C3—C2—H2	109.3	H9A—C9—H9C	109.5
C1—C2—H2	109.3	H9B—C9—H9C	109.5
C5—C2—H2	109.3	C11—C10—C15	117.4 (3)
O3—C3—C4	121.1 (3)	C11—C10—C1	121.3 (2)
O3—C3—C2	119.5 (3)	C15—C10—C1	121.3 (3)
C4—C3—C2	119.3 (3)	C10—C11—C12	121.6 (3)
C3—C4—H4A	109.5	C10—C11—H11	119.2
C3—C4—H4B	109.5	C12—C11—H11	119.2
H4A—C4—H4B	109.5	C13—C12—C11	119.4 (4)
C3—C4—H4C	109.5	C13—C12—H12	120.3
H4A—C4—H4C	109.5	C11—C12—H12	120.3
H4B—C4—H4C	109.5	C14—C13—C12	120.4 (4)
O4—C5—C6	121.7 (3)	C14—C13—H13	119.8
O4—C5—C2	119.8 (3)	C12—C13—H13	119.8
C6—C5—C2	118.5 (3)	C13—C14—C15	119.5 (4)
C5—C6—H6A	109.5	C13—C14—H14	120.2
C5—C6—H6B	109.5	C15—C14—H14	120.2
H6A—C6—H6B	109.5	C14—C15—C10	121.6 (3)
C5—C6—H6C	109.5	C14—C15—H15	119.2
H6A—C6—H6C	109.5	C10—C15—H15	119.2
H6B—C6—H6C	109.5		

### Hydrogen-bond geometry (Å, º)

**Table:**

*D*—H···*A*	*D*—H	H···*A*	*D*···*A*	*D*—H···*A*
N1—H1···O2^i^	0.97 (3)	2.26 (3)	3.180 (4)	158 (2)

Symmetry code: (i) *x*+1, *y*, *z*.
